# Medical Debts

**Published:** 1897-04-17

**Authors:** 


### MEDICAL DEBTS.

Dr. Hugh Woods, hon. secretary of the London and Counties Medical Protection Society, writes : Several complaints having reached my society concerning the methods adopted by certain so-called associations professing to have been formed for the purpose of collecting medical debts, my society will be glad to hear from any of your readers who have had dealings with any of such associations, and who are dissatisfied with their procedure.

